# Changes in central venous-to-arterial carbon dioxide tension induced by fluid bolus in critically ill patients

**DOI:** 10.1371/journal.pone.0257314

**Published:** 2021-09-10

**Authors:** Charalampos Pierrakos, David De Bels, Thomas Nguyen, Dimitrios Velissaris, Rachid Attou, Jacques Devriendt, Patrick M. Honore, Fabio Silvio Taccone, Daniel De Backer

**Affiliations:** 1 Intensive Care Department, Brugmann University Hospital, Université Libre de Bruxelles, Bruxelles, Belgium; 2 Internal Medicine Department, University Hospital of Patras, Patras, Greece; 3 Intensive Care Department, Erasme Hospital, Université Libre de Bruxelles, Brussels, Belgium; 4 Department of Intensive Care, CHIREC Hospitals, Université Libre de Bruxelles, Bruxelles, Belgium; Scuola Superiore Sant’Anna, ITALY

## Abstract

**Background:**

In this prospective observational study, we evaluated the effects of fluid bolus (FB) on venous-to-arterial carbon dioxide tension (P_va_CO_2_) in 42 adult critically ill patients with pre-infusion P_va_CO_2_ > 6 mmHg.

**Results:**

FB caused a decrease in P_va_CO_2_, from 8.7 [7.6−10.9] mmHg to 6.9 [5.8−8.6] mmHg (p < 0.01). P_va_CO_2_ decreased independently of pre-infusion cardiac index and P_va_CO_2_ changes during FB were not correlated with changes in central venous oxygen saturation (S_cv_O_2_) whatever pre-infusion CI. Pre-infusion levels of P_va_CO_2_ were inversely correlated with decreases in P_va_CO_2_ during FB and a pre-infusion P_va_CO_2_ value < 7.7 mmHg could exclude a decrease in P_va_CO_2_ during FB (AUC: 0.79, 95%CI 0.64–0.93; Sensitivity, 91%; Specificity, 55%; p < 0.01).

**Conclusions:**

Fluid bolus decreased abnormal P_va_CO_2_ levels independently of pre-infusion CI. Low baseline P_va_CO_2_ values suggest that a positive response to FB is unlikely.

## Introduction

The venous-to-arterial carbon dioxide tension difference is an easily-derived metabolic index that can be used to assess the adequacy of tissue perfusion to support the body’s metabolism [1–3]. Applying Fick’s formula for CO_2_ shows that the difference between the mixed venous and arterial CO_2_ content equals the ratio between CO_2_ production (VCO_2_) and cardiac output. As CO_2_ content is difficult to assess, it can be replaced with the partial pressure of CO_2_ in the blood, since there is a linear relationship between these two parameters, at least in a large physiological range [4]. Ideally, venous-to-arterial carbon dioxide tension difference should be derived using pulmonary artery obtained PCO_2_. Nevertheless, Swan–Ganz catheter is not used often in contemporary intensive care [5]. Central venous venous-to-arterial carbon dioxide tension (P_va_CO_2_) even though is not interchangeable to mixed venous [6] can be used instead as a high P_va_CO_2_ (> 6 mmHg) indicates that tissue perfusion is not sufficiently high to remove the CO_2_ produced by the tissues [7]. Of note, persistent abnormal P_va_CO_2_ levels can be related to poor outcome in critically ill patients [8, 9]. Accordingly, P_va_CO_2_ might be an interesting target for resuscitation [10].

Unfortunately, the interventions potentially improving P_va_CO_2_ have not yet been adequately evaluated. Observational studies have shown that resuscitation maneuvers improving central venous saturation and arterial pressure might not be related to a decrease in P_va_CO_2_ [6, 11, 12]. Dobutamine can cause a decrease in P_va_CO_2_ due to an increase in CI, although a paradoxical increase might be observed at higher doses [13, 14]. Fluid bolus (FB) might be another therapeutic option in patients with abnormal P_va_CO_2_. Mecher et al. reported that FB decreased high P_va_CO_2_ in patients with septic shock, but the authors enrolled only septic patients with low CI [15]. As elevated P_va_CO_2_ might also represent microcirculatory alterations in the context of preserved CI [16], one may wonder whether FB decreases P_va_CO_2_ independently of the baseline CI.

The aim of this study was to investigate whether FB decreases P_va_CO_2_ and to determine their relationships with CI and oxygenation changes.

## Methods

### Design and setting

In this prospective observational study, we collected data from patients treated in Brugmann University Hospital’s 33-bed intensive care unit in Brussels between January and June 2015. Approval was obtained from the Ethics Committee (CE2014/122) of CHU-Brugmann.

### Inclusion and exclusion criteria

Patients with P_va_CO_2_ > 6 mmHg in whom the attending physician decided for a FB of either colloids or crystalloids within 30–40 min at any time of their stay in the ICU were considered eligible for this study. We included patients using a deferred informed consent as FB was part of standard treatment and we used not invasive methods for monitoring. Informed consent was obtained from all patients or, when that was not feasible, a consent form was gathered from the next-of-kin as soon as possible after FB but before ICU discharge.

Each patient was assessed once. The exclusion criteria were: 1) patients younger than 18 years old; 2) not equipped with jugular or subclavian venous catheter and arterial catheter; 3) measurement of cardiac output with cardiac ultrasound was not possible due to lack of acoustic window; 4) patients receiving extracorporeal membrane oxygenation (ECMO) support; 5) PCO_2_ higher than 75 mmHg in venous or arterial blood gas analysis; 6) atrial fibrillation; 7) other simultaneous interventions (i.e., introduction or increase in inotrope dosage, mode changes, or the introduction of mechanical ventilation) within 30 min prior to fluid administration.

### Data and sample collections

Demographics, the type of fluids used for FB, clinical data concerning treatment (mechanical ventilation, inotropic agents), and laboratory data were collected for each patient. The Acute Physiology and Chronic Health Evaluation (APACHE) II score were used to assess the severity of disease at the time of inclusion in the study.

Using Doppler transthoracic echocardiography (GE Healthcare Vivid S5), we measured the left ventricular outflow tract (LVOT) blood velocity time integral (VTI) just prior to the administration of FB. To calculate stroke volume (SV) and CI, LVOT diameter was measured below the aortic valve at the aortic cusp insertion points in the parasternal long-axis view. Immediately after FB, we repeated the measurements. Both measurements were stored and analyzed off-line. Three consecutive velocity curves were measured, and the average VTI was calculated. We used the same value of LVOT diameter to calculate SV and CI before and after FB. Each patient was assessed once. No interventions were allowed during fluid administration.

Arterial and central venous blood gas analysis were simultaneously obtained just before and after FB. We measured the haemoglobin, arterial, and venous oxygen tensions (P_a_O_2_ and P_v_O_2_, respectively) and oxygen saturation (S_a_O_2_ and S_cv_O_2_). Applying the usual formulas, we calculated the arterial (C_a_O_2_) and venous (C_v_O_2_) oxygen content and oxygen delivery (DO_2_), and oxygen consumption (VO_2_). The P_va_CO_2_ and P_va_CO_2_/C_av_O_2_ ratios were calculated before and after FB.

### Diagnostic definitions

All the diagnostic definitions were set beforehand. The smallest detectable difference (SDD) of P_va_CO_2_ was expected to be ±2.06 mm Hg as it was evaluated in a previous study in critically ill patients [17]. Accordingly, patients were considered as ‘P_va_CO_2_ responders’ if they had a decrease in P_va_CO_2_ > 2 mmHg. ‘Fluid responders’ were defined as patients who had an increase in CI > 15% [18]. Sepsis was defined according to standard criteria [19]. As changes in P_va_CO_2_ may be affected by baseline value and as P_va_CO_2_ is inversely related to cardiac index, we separated patients into ‘low’ and ‘high’ cardiac index using a cut-off value of 2.2 L/min/m^2^, similarly to a previous study [15]. Of note, the term ‘low CI’ should not be misinterpreted as in some case the low CI may still be adequate [20].

### Primary outcome

The primary endpoint was to evaluate whether FB can decrease P_va_CO_2_ by at least 2 mmHg on average.

### Secondary outcomes

The secondary endpoint was to investigate changes of P_va_CO_2_ during FB in patients with baseline CI less or more 2.2 L/min/m^2^ and its relationship with changes of CI and S_cv_O_2_. The value of baseline P_va_CO_2_ for the prediction of a decrease in P_va_CO_2_ during FB will be evaluated.

### Statistical analysis

We performed statistical analysis using R through the R-studio interface (www.r-project.org, R version 3.3.1). We used a Kolmogorov-Smirnov test to verify the normality of the distribution of the continuous variables. Normally distributed and non-normally distributed data were compared using a Student’s t-test or Wilcoxon signed-rank test, as appropriate. Categorical variables were compared using Fisher’s exact test. Pearson correlation and scatter diagrams were used to assess correlations between values. Univariate regression analysis was performed to evaluate the association between decrease P_va_CO_2_ > 2mmHg and baseline CI, fluid type and mechanical ventilation. Receiver operating characteristics (ROC) analysis was used to derive the prognostic discriminatory performance of baseline P_va_CO_2_ in determining a decrease of P_va_CO_2_ during FB. The sample size was calculated to aim for an AUC of greater than 0.8, which is usually considered as having a good predictive ability. Assuming a fluid responsiveness rate of 30% in mixed population of critically ill patients [21] 40 patients were required to obtain 90% power (alpha 0.05). The Youden index was used to derive the optimal cut-off. Statistical significance was defined as p < 0.05.

## Results

We evaluated 80 patients who received FB during the study period. Two patients refused to give informed consent and were excluded from any further analysis. Forty-two patients (73 years (64−83) and APACHE II score on admission 21(15−29)) met our entry criteria (S1 Fig). Twenty-four of the patients (57%) received colloids (Geloplasma^®^, Fresenius-Kabi AG, Bad Homburg, Germany) and 18 (43%) crystalloids (Plasma-Lyte A, Baxter Healthcare, Deerfield, IL) (S1 Table). The median given volume was 6.3 ml/kg [6.3−7.1] for FB with colloids and 14.9 ml/kg [12.1−19.6] for FB with crystalloids within a median time of 33 min [27− 44]. Central venous pressure increased after FB from 8.5 mmHg [4.0−11.2] to 11.1 mmHg [9.2− 13.0] (p<0.01). No differences were observed in the increases in central venous pressure after FB between the patients who received colloids or crystalloids (33% [30–73] vs 22% [9−44], p = 0.07). Fourteen patients (33%) had an increase in CI >15% after FB. No differences were observed in the changes in CI after FB between the patients who received colloids or crystalloids (13% [0−21]vs 12% [2−25], p = 0.22). Nineteen (45%) of the patients were supported with mechanical ventilation during FB, and 11 (26%) were under sedation. No changes in respiratory rate were observed during FB (21 ± 5 resp/min to 21 ± 6 resp/min, p = 0.92). Sixteen patients had a CI ≤ of 2.2 L/min/m^2^ before FB, and 26 had a CI of > 2.2 L/min/m^2^.

### Primary outcome

**Fig 1:**
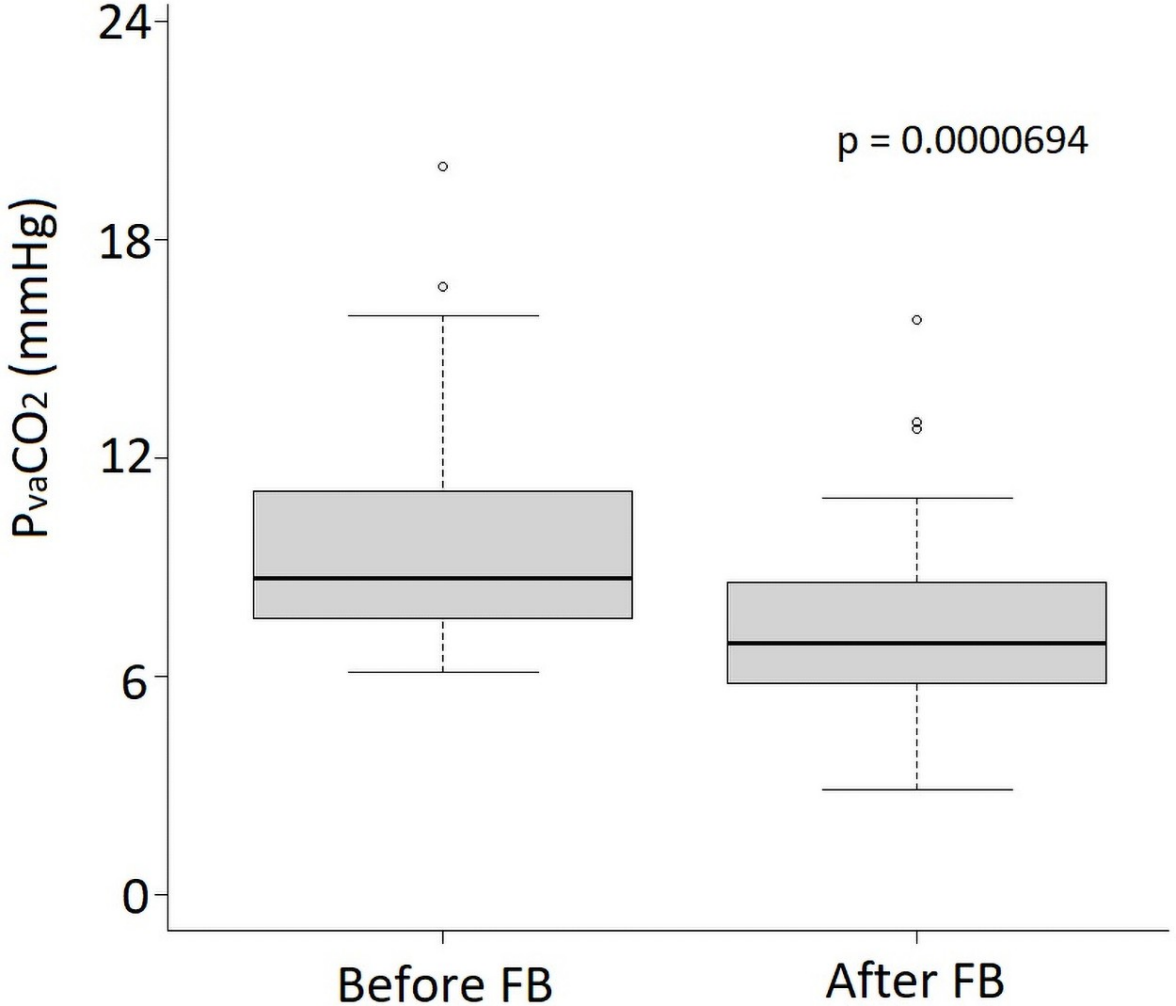
Evolution of central venous-to-arterial carbon dioxide tension difference (P_va_CO_2_) during fluid bolus.

The median P_va_CO_2_ before FB was 8.7 [7.6−10.9] and did not differ between intubated and not intubated patients (9.2 [7.7−13.5] mmHg versus 8.4 [7.4−10.2] mmHg; p = 0.23). FB decreased P_va_CO_2_ to 6.9 [5.8−8.6] mmHg (p < 0.01) (Fig 1). Twenty-two patients (52%) had a decrease in P_va_CO_2_ > 2 mmHg (‘P_va_CO_2_ responders’). The hemodynamic and metabolic characteristics of the patients, as well as their changes, are presented in Table 1 and S2 Table. ‘P_va_CO_2_ responders’ had a higher relative and absolute increase in CI compared to ‘P_va_CO_2_ non-responders’.

**Table 1:** Patients’ baseline hemodynamic and metabolic variables and changes during fluid bolus according to a decrease (or not) in central venous-to-arterial carbon dioxide tension difference (P_va_CO_2_) > 2 mmHg (P_va_CO_2_ non-responders and responders). Changes are presented as relative (d, %) and absolute values (Δ). Values are presented either as means with standard deviations (±) or as median values and percentiles 25 and 75.

	P_va_CO_2_ non-responders	P_va_CO_2_ responders	*p values*
No of patients	20	22	
**Baseline hemodynamic variables**			
Mean arterial pressure (mmHg)	71 ± 15	82 ± 14	<0.01
Pulse Pressure (mmHg)	52 ± 16	58 ± 16	0.23
Central Venous Pressure (mmHg)	8 ± 5	8 ± 4	0.28
Velocity Time Integral (cm)	14.7 ± 5	13.6 ± 5	0.91
Stroke Volume (ml)	55 ± 22	51 ± 21	0.54
Heart Rate (beats /min)	87 ± 20	97 ± 19	0.13
Cardiac Index (L/min/m^2^)	2.7 (1.7−3.3)	2.6 (1.9−3.2)	0.77
**Baseline metabolic variables**			
Oxygen delivery (mL/min/m^2^)	326 (287−442)	416 (290−472)	0.28
S_cv_O_2_ (%)	61 ± 11	65 ± 8	0.17
Oxygen consumption (mL/min/m^2^)	116 (95−140)	122 (97−159)	0.65
Oxygen extraction (%)	33 (26−46)	33 (29−37)	0.44
P_va_CO_2_(mmHg)	8.2 (6.7−9.7)	10.1 (8.7−12)	<0.01
P_va_CO_2_/ C_av_O_2_	1.7 (1.4−2.1)	2.1 (1.7−2.6)	<0.01
Lactate (mmol/L)	1.9 (1.6−3.1)	2.1 (1.6−3.7)	0.47
**Hemodynamic variable changes during FB**			
Δ Mean arterial pressure (mmHg)	2 (-2−9)	4 (-4−10)	0.99
d Mean arterial pressure (%)	3 (-3−13)	5 (-4−12)	0.93
Δ Pulse Pressure (mmHg)	1 (-3−15)	5 (-3−17)	0.97
d Pulse Pressure (%)	2 (-6−33)	10 (-4−24)	0.86
Δ Central Venous Pressure (mmHg)	2 (1−4)	3 (1−4)	0.99
d Central Venous Pressure (%)	16 (12–32)	27 (10–50)	0.78
Δ Velocity Time Integral (cm)	0 (-1−4)	3 (2−4)	0.03
d Velocity Time Integral (%)	5 (-2−19)	21 (14−40)	<0.01
Δ Stroke Volume (ml)	2 (-1−13)	11 (7−17)	0.02
d Stroke Volume (%)	5 (-2−19)	21 (14−40)	<0.01
Δ Heart Rate (beats/min)	-2 (-11−1)	-3 (-6−1)	0.88
d Heart Rate (%)	-2 (-12−2)	-2 (-6−1)	0.81
Δ Cardiac Index (L/min/m2)	0.1 (-0.1−0.4)	0.5 (0.4−0.7)	<0.01
d Cardiac Index (%)	6 (1−13)	19 (11−40)	<0.01
**Metabolic variable changes during FB**			
Δ Oxygen delivery (mL/min/m2)	-25 (-28−13)	46 (-19−97)	0.02
d Oxygen delivery (%)	-7 (-9−5)	10 (-6−32)	0.02
Δ ScvO2 (%)	-1 (-3−3)	1 (-1−4)	0.41
Δ Oxygen extraction (%)	1 (-3−4)	-1 (-3−0)	0.16
Δ Oxygen consumption (mL/min/m2)	1 (-6−15)	4 (-18−32)	0.29
d Oxygen consumption (%)	0 (-9−12)	5 (-7−32)	0.19
Δ PvaCO2(mmHg)	0 (-1−1)	-4 (-5−-3)	<0.01
d PvaCO2 (%)	-4 (-9−10)	-40 (-48−-30)	<0.01
Δ PvaCO2/ CavO2	0.02 (-0.04−0.5)	-0.6 (-0.9−-0.4)	<0.01
d PvaCO2/ CavO2 (%)	2 (-2−33)	-33(-37−-25)	<0.01

**S**_**cv**_**O**_**2**_: central venous oxygen saturation, **P**_**va**_**CO**_**2**_: venous-to-arterial carbon dioxide tension, **C**_**av**_**O**_**2**_: arterial-venous oxygen content difference.

There was no association between the decrease of P_va_CO_2_ > 2 mmHg after FB and the pre-infusion levels of CI (i.e. ‘low’ or ‘high CI’). Additionally, the type of fluid used for FB and mechanical ventilation were not found to be associated with the likelihood of decreasing P_va_CO_2_ > 2 mmHg after FB (S3 Table).

### Secondary outcomes

**Fig 2:**
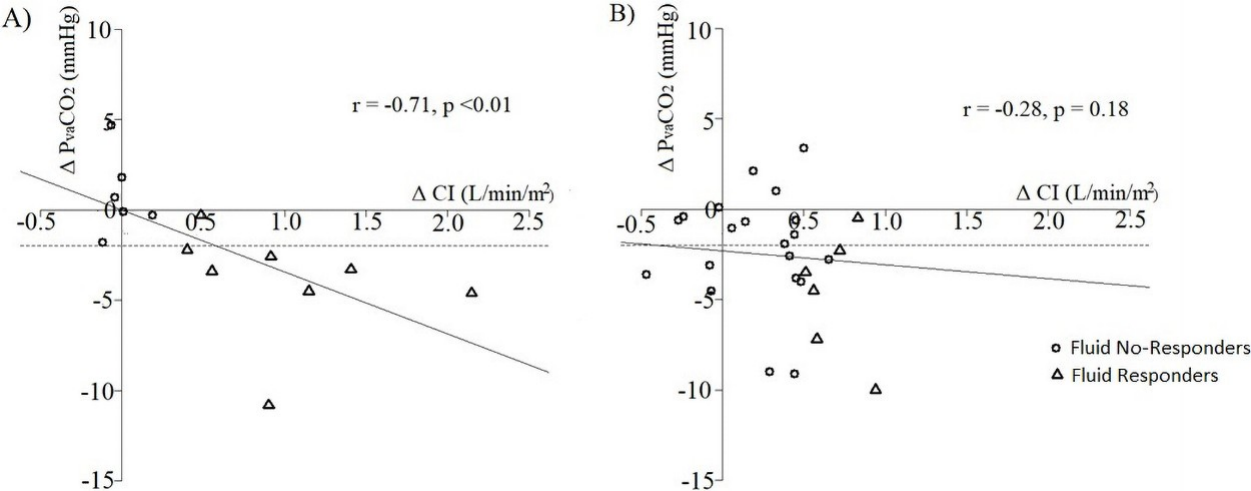
Relationship between absolute changes in P_va_CO_2_ (Δ P_va_CO_2_) during fluid bolus and absolute changes in cardiac index (Δ CI). Panel A: Patients with CI ≤ 2.2 L/min/m^2^; Panel B: Patients with CI > 2.2 L/min/m^2^. d CI: relative to baseline values changes in CI. The horizontal dotted line corresponds to Δ P_va_CO_2_−2 mmHg. Triangle points represent “Fluid responders” (d CI > 15%) and circle points “Fluid non-responders” (d CI ≤ 15%).

A correlation between changes in CI and P_va_CO_2_ was observed only in patients who had a low CI before FB (r = -0.71, p < 0.01). None of the patients who had an increase in CI > 15% (Fig 2) experienced an increase in P_va_CO_2_. Because estimation of the area of LVOT represents the major source of error in calculating cardiac output with transthoracic echocardiography [22] we repeated the analysis using only VTI: similar results were found when P_va_CO_2_ changes were assessed with changes in VTI (S2 Fig).

We found no statistically significant correlation between P_va_CO_2_ and S_cv_O_2_ changes, independently of the baseline CI. S_cv_O_2_ could either increase, decrease, or remained unchanged in ‘P_va_CO_2_ responders’ (S3 Fig).

**Fig 3:**
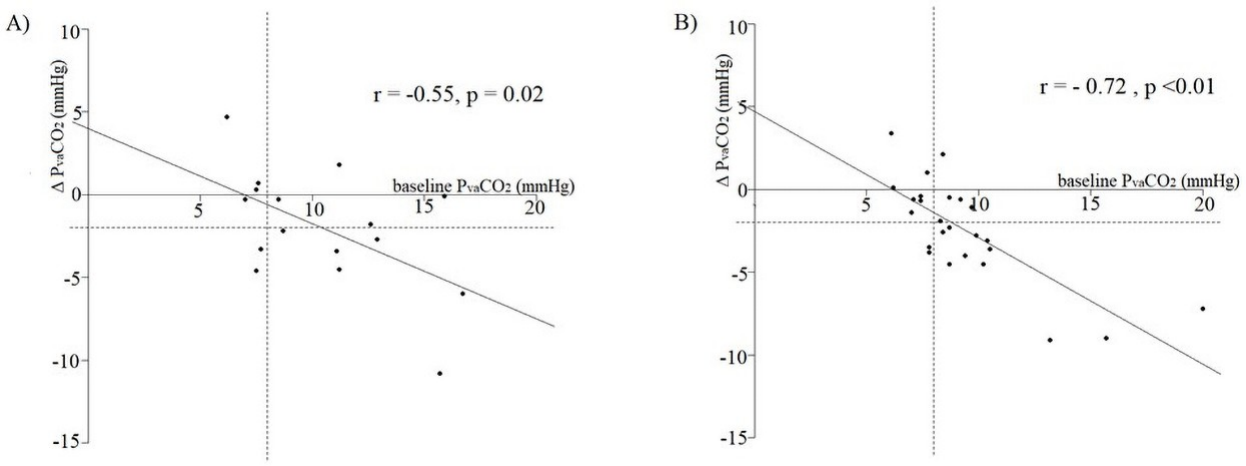
Relationship between baseline P_va_CO_2_ and changes in P_va_CO_2_ (Δ P_va_CO_2_) during fluid bolus. Panel A: Patients with CI ≤ 2.2 L/min/m^2^; Panel B: Patients with CI > 2.2 L/min/m^2^. The vertical dotted line corresponds to the baseline P_va_CO_2_ 8mmHg. The horizontal dotted line corresponds to Δ P_va_CO_2_−2 mmHg.

A value < 7.7 mmHg could exclude a decrease of P_va_CO_2_ during the FB, independently of baseline CI (AUC: 0.79, 95%CI 0.64 ‒ 0.93; Sensitivity, 91%; Specificity, 55%; p < 0.01) (S4 Fig). Baseline P_va_CO_2_ was correlated with the changes in P_va_CO_2_ during FB in patients with low as well as in patients with high CI before FB (low CI before FB: r = -0.55, p = 0.02, high CI before FB: r = -0.72, p < 0.01) (Fig 3).

## Discussion

The results of this study can be summarized as follows: 1) FB can decrease an abnormal high P_va_CO_2_ in critically ill patients independently of the before FB CI values, 2) the response of P_va_CO_2_ to FB is highly variable, yet low baseline P_va_CO_2_ (6 to 8 mmHg) can exclude a positive response.

The clinical implication of the study is that P_va_CO_2_ derived from central venous and arterial blood gas analysis can be used in clinical practice for the evaluation of FB response (S5 Fig). ‘P_va_CO_2_ responders’ had a significantly higher increase in CI, which confirms that the CI augmentation is implicated in the decrease in P_va_CO_2_ during FB. Of note, in theory, FB can cause an increase in P_va_CO_2_ due to acute decrease in hemoglobin concentration [23]. The results of our study showed that an increase in P_va_CO_2_ is rare after FB. Notwithstanding, given that none of the ‘CI responders’ presented an increase in P_va_CO_2_ can be considered as an adverse effect of FB and it can be used as a safety limit for FB in case no CI monitoring is available.

Interestingly, the group of ‘P_va_CO_2_ responders’ was not exactly the same as ‘CI responders’: several ‘CI responders’ did not have a decrease in P_va_CO_2_, whereas P_va_CO_2_ decreases were not always associated with increases in CI >15%. Similar observations were reported by other teams using various measurements related to tissue perfusion [24–26]. Different factors can explain this phenomenon. Increases in CI after FB might not always lead to an improvement in tissue perfusion [27], particularly when CI is not a major contributing factor for microcirculatory abnormalities. Additionally, in patients with high CI changes in P_va_CO_2_ are expected to be limited as the relationship between these two variables is curvilinear [28]. Of note, we detected a statistically significant correlation of CI changes with P_va_CO_2_ only in the group of patients with low baseline CI. Furthermore, ‘CI responders’ are defined based on relative changes in CI. Accordingly, several patients with increases in CI between 0.4–0.5 L/min/m^2^ were allocated as ‘CI non-responders’. Moreover, evaluation of changes in CI with the method of cardiac echocardiography might not be precise in detecting mild changes [29].

The results of this study add to our knowledge of the optimization of fluid administration in critically ill patients using P_va_CO_2_ values. Recognition of the severity of inadequate tissue perfusion based on the levels of P_va_CO_2_ can guide the physician to decide fluid administration: a low P_va_CO_2_ can be considered as an indication to avoid FB whereas a high level may not always be an indication for FB evaluation of its effects is required. This finding is in line with the results of previous studies, which showed mild microcirculation abnormalities are less likely to be improved after FB [24]. Nevertheless, some patients with high P_va_CO_2_ failed to respond to FB, and therefore, the decision for FB administration should not be based only on the P_va_CO_2_ levels. Furthermore, whether FB is the more appropriate treatment for the treatment of high P_va_CO_2_ levels compared to other interventions aiming to improve tissue perfusion (e.g dobutamine, nitrate) should be further evaluated in future studies.

P_va_CO_2_ changes were not found to be correlated to S_cv_O_2_. The meaning of this finding is dual. First, P_va_CO_2_ changes after FB potentially can provide additional information to S_cv_O_2_. As in other studies, P_va_CO_2_ may remain altered when S_cv_O_2_ is close to normal, so that P_va_CO_2_ can be used in addition to S_cv_O_2_ for evaluating the adequacy of resuscitation in critically ill patients [30–32]. P_va_CO_2_ is related to tissue perfusion independently of the presence of tissue hypoxia [3], whereas S_cv_O_2_ reflects the balance between oxygen delivery and oxygen consumption [33]. In the majority of patients, improvement in tissue perfusion (‘P_va_CO_2_ responders’) was associated with an increase in S_cv_O_2_. However, increases in S_cv_O_2_ occurred in some ‘P_va_CO_2_ non-responders’. As multiple patterns were observed, our study underscores the multiple factors implicated in changes in P_va_CO_2_ and S_cv_O_2_ after FB. Second, the absence of correlation between P_va_CO_2_ changes and S_cv_O_2_ suggests that the Haldane effect has only a minor impact on the changes of P_va_CO_2_ during fluid bolus. Given that arterial saturation and PCO_2_ did not change in our cohort increases in S_cv_O_2_ secondary to a positive fluid response could cause an increase in venous partial pressure of CO_2_ and consequently an increase in P_va_CO_2_ [34].

The strength of this study is that we assessed the effect of FB on P_va_CO_2_ in a non-selected critically ill population with abnormal high P_va_CO_2_. The high range of the pre-infusion CI permitted the study of P_va_CO_2_ changes after FB in a diversity of hemodynamic conditions, whereas no respiratory variations or other interventions can explain these changes. Nevertheless, this study has several limitations. First, we assessed only acute changes in P_va_CO_2_ so that we cannot ensure that these beneficial effects were maintained. However, evaluation of P_va_CO_2_ over several hours might be challenging as metabolic changes can also occur, especially in non-sedated patients, in addition to other cardiovascular events. Second, metabolic changes independent of FB may have occurred. However, major spontaneous metabolic changes are not expected to occur during the short observational period of the study. Third, only central venous and not mixed venous-to-arterial carbon dioxide tension differences were evaluated. Fourth, we did not investigate thoroughly the effects of other therapeutic interventions (e.g. mechanical ventilation, inotropes) on P_va_CO_2_ as well as its changes during FB.

### Conclusions

Abnormal high P_va_CO_2_ can be decreased with FB independently of the levels of the pre-infusion CI. A decrease in P_va_CO_2_ after FB is unlikely in patients with pre-infusion P_va_CO_2_ below 7.7 mmHg. Increases in P_va_CO_2_ can be considered as an indication of negative response to FB. Decreases in P_va_CO_2_can be considered a positive response to FB, even though they might not always be associated with relative increases in CI >15%. Changes in CI can only partially explain decreases in P_va_CO_2_. P_va_CO_2_ and S_cv_O_2_ provide complementary information for the effects of FB on tissue perfusion.

## Supporting information

S1 Fig Flowchart of patients selection. (PDF)

S2 Fig Relationship between changes in P_va_CO_2_ (Δ P_va_CO_2_) during fluid bolus and absolute changes in velocity time integral (Δ VTI). Panel A: Patients with CI ≤ 2.2 L/min/m2; Panel B: Patients with CI > 2.2 L/min/m2. d VTI: relative to baseline values changes in VTI. Horizontal dotted line corresponds to Δ P_va_CO_2_−2 mmHg.
(PDF)

S3 Fig Relationship between changes in central venous oxygen saturation (ScvO2) during fluid bolus and absolute changes in P_va_CO_2_ (Δ P_va_CO_2_). Panel A: Patients with CI ≤ 2.2 L/min/m2; Panel B: Patients with CI > 2.2 L/min/m2. d CI: relative to baseline values changes in CI. Vertical dotted line corresponds to Δ P_va_CO_2_−2 mmHg.
(PDF)

S4 Fig ROC curve for baseline values of P_va_CO_2_ for prediction of P_va_CO_2_ decrease during fluid bolus. (PDF)

S5 Fig Algorithm of interpretation P_va_CO_2_ in relation to decision and appreciation of fluid bolus. (PDF)

S1 Table Characteristics of the patients received fluid bolus (FB) and included in the study. (PDF)

S2 Table Blood gas analysis derived parameters before and after fluid bolus. (PDF)

S3 Table Univariate logistic regression analysis with positive P_va_CO_2_ decrease > 2mmHg after fluid bolus as the dependent variable. (PDF)
