# Supplementary material for: Changes in central venous-to-arterial carbon dioxide tension induced by fluid bolus in critically ill patients
Source: PLoS One. 2021 Sep 10;16(9):e0257314. doi: 10.1371/journal.pone.0257314 (PMC8432848; doi:10.1371/journal.pone.0257314)

S2 Fig . Relationship between changes in $P_{va}CO_2$ (Δ $P_{va}CO_2$) during fluid bolus and absolute changes in velocity time integral (Δ VTI). Panel A: Patients with CI $\leq$ 2.2 L/min/m^2 ; Panel B: Patients with CI > 2.2 L/min/m^2. d VTI: relative to baseline values changes in VTI. Horizontal dotted line corresponds to Δ $P_{va}CO_2$ -2 mmHg.

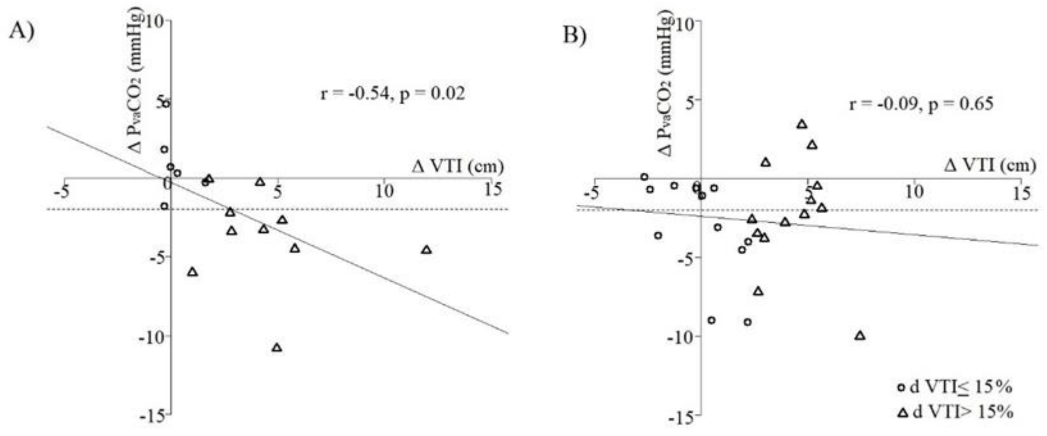

Supplement: S2 Fig — Panel A: Patients with CI ≤ 2.2 L/min/m2; Panel B: Patients with CI > 2.2 L/min/m2. d VTI: relative to baseline values changes in VTI. Horizontal dotted line corresponds to Δ PvaCO2−2 mmHg. (PDF) [file pone.0257314.s002.pdf]
