# Supplementary material for: Changes in central venous-to-arterial carbon dioxide tension induced by fluid bolus in critically ill patients
Source: PLoS One. 2021 Sep 10;16(9):e0257314. doi: 10.1371/journal.pone.0257314 (PMC8432848; doi:10.1371/journal.pone.0257314)

S3 Fig. Relationship between changes in central venous oxygen saturation (ScvO2) during fluid bolus and absolute changes in PvaCO2 (Δ PvaCO2). Panel A: Patients with CI ≤ 2.2 L/min/m^2 ; Panel B: Patients with CI > 2.2 L/min/m^2. d CI: relative to baseline values changes in CI. Vertical dotted line corresponds to Δ $P_{va}CO_2$ -2 mmHg.

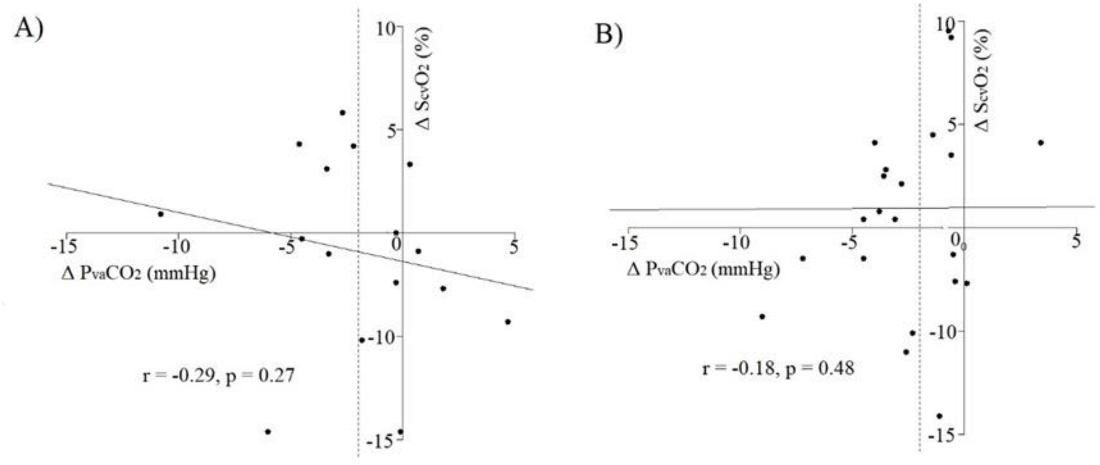

Supplement: S3 Fig — Panel A: Patients with CI ≤ 2.2 L/min/m2; Panel B: Patients with CI > 2.2 L/min/m2. d CI: relative to baseline values changes in CI. Vertical dotted line corresponds to Δ PvaCO2−2 mmHg. (PDF) [file pone.0257314.s003.pdf]
