# Supplementary material for: Changes in central venous-to-arterial carbon dioxide tension induced by fluid bolus in critically ill patients
Source: PLoS One. 2021 Sep 10;16(9):e0257314. doi: 10.1371/journal.pone.0257314 (PMC8432848; doi:10.1371/journal.pone.0257314)

S4 Fig. ROC curve for baseline values of $P_{va}CO_2$ for prediction of $P_{va}CO_2$ decrease during fluid bolus .

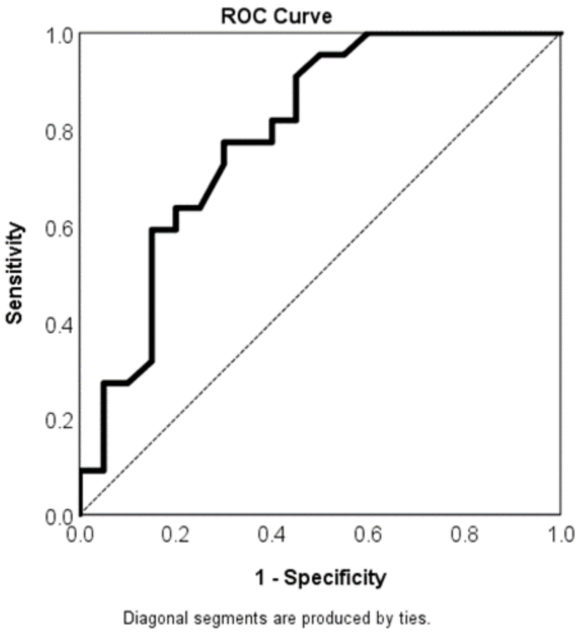

Supplement: S4 Fig — (PDF) [file pone.0257314.s004.pdf]
