# Supplementary material for: Changes in central venous-to-arterial carbon dioxide tension induced by fluid bolus in critically ill patients
Source: PLoS One. 2021 Sep 10;16(9):e0257314. doi: 10.1371/journal.pone.0257314 (PMC8432848; doi:10.1371/journal.pone.0257314)

S5 Fig. Algorithm of interpretation $P_{va}CO_2$ in relation to decision and appreciation of fluid bolus.

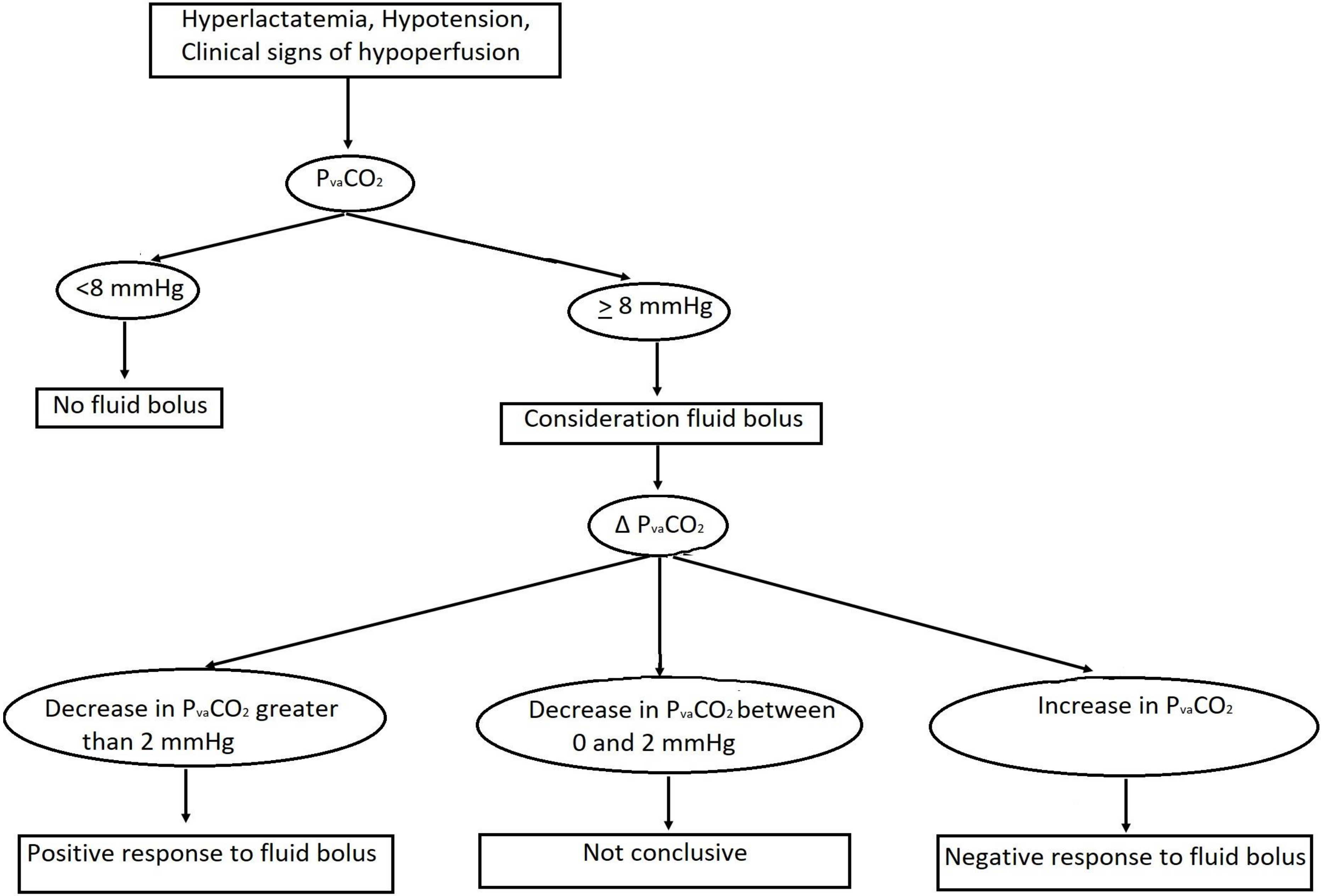

Supplement: S5 Fig — (PDF) [file pone.0257314.s005.pdf]
