# Supplementary material for: Changes in central venous-to-arterial carbon dioxide tension induced by fluid bolus in critically ill patients
Source: PLoS One. 2021 Sep 10;16(9):e0257314. doi: 10.1371/journal.pone.0257314 (PMC8432848; doi:10.1371/journal.pone.0257314)
Supplement: S1 Table — (PDF) [file pone.0257314.s006.pdf]

S1 Table. Characteristics of the patients received fluid bolus (FB) and included in the study.

**No of patients**		42
**Age**		73 (64−83)
**APACHE II**		21(15−29)
**Diagnosis**		
	**Sepsis (%)**	20 (47)
	**Hypovolemia (%)**	14 (33)
	**Cardiac dysfunction (%)**	7 (16)
**Noradrenalin support >0.1μcg/kg/min**		12 (28)
**Mechanical ventilation (%)**		19 (45)
**FB with Colloids (%)**		24 (57)
**Principal reason for FB (%)**		
	**High levels of lactate**	21 (50)
	**Hypotension**	10 (24)
	**Oliguria or clinical signs of hypoperfusion**	11 (26)
