# Supplementary material for: Changes in central venous-to-arterial carbon dioxide tension induced by fluid bolus in critically ill patients
Source: PLoS One. 2021 Sep 10;16(9):e0257314. doi: 10.1371/journal.pone.0257314 (PMC8432848; doi:10.1371/journal.pone.0257314)
Supplement: S2 Table — (PDF) [file pone.0257314.s007.pdf]

S2 Table. Blood gas analysis derived parameters before and after fluid bolus

	Before FB	After FB	p value
**$P_{va}CO_2$ non-responders**			
**Arterial pH**	7.42 (7.36− 7.45)	7.41 (7.37−7.45)	0.84
**Venous pH**	7.37 (7.30 −7.39)	7.36 (7.31− 7.41)	0.99
**P_aCO_2**	35 (31− 38)	34 (32− 37)	0.18
**P_vCO_2**	43 (39 −48)	44 (41 −46)	0.39
**Hemoglobin**	10.1 (8.7 −13.1)	9.2 (7.8 −12.1)	<0.01
**S_aO_2**	96 (94 −96)	96 (95− 97)	0.23
**$P_{va}CO_2$ responders**			
**Arterial pH**	7.39(7.34− 7.43)	7.39 (7.35− 7.42)	0.91
**Venous pH**	7.33 (7.28− 7.37)	7.34(7.29 −7.38)	0.26
**P_aCO_2**	36(31− 43)	35 (33− 45)	0.09
**P_vCO_2**	46 (41− 55)	43 (39− 50)	<0.01
**Hemoglobin**	11.9 (10.8− 12.4)	10.9 (9.6 −11.8)	<0.01
**S_aO_2**	97(94 −98)	97 (95 −98)	0.46

pH: potential hydrogen; **P_aCO_2** : partial pressure of carbon dioxide in arterial blood sample; **P_vCO_2**: partial pressure of carbon dioxide in venous blood sample; **S_aO_2**: oxygen saturation measured in arterial blood sample
