# Supplementary material for: Changes in central venous-to-arterial carbon dioxide tension induced by fluid bolus in critically ill patients
Source: PLoS One. 2021 Sep 10;16(9):e0257314. doi: 10.1371/journal.pone.0257314 (PMC8432848; doi:10.1371/journal.pone.0257314)
Supplement: S3 Table — (PDF) [file pone.0257314.s008.pdf]

S3 Table. Univariate logistic regression analysis with positive $P_{va}CO_2$ decrease > 2mmHg after fluid bolus as the dependent variable

	**Reference Group**	**Odds Ratio (95% CI)**	**p Value**
**"Low" Cardiac Index**	"High" Cardiac Index	1.4 (0.35−5.34)	0.64
**Mechanical ventilation**	No mechanical ventilation	0.5 (0.13−1.92)	0.32
**Colloids Fluid Bolus**	Crystalloids Fluid Bolus	1.3 (0.32−4.97)	0.73
