# Supplementary material for: Changes in central venous-to-arterial carbon dioxide tension induced by fluid bolus in critically ill patients
Source: PLoS One. 2021 Sep 10;16(9):e0257314. doi: 10.1371/journal.pone.0257314 (PMC8432848; doi:10.1371/journal.pone.0257314)
Supplement: Attachment — Submitted filename: Reviewers_comments_18821_f.docx [file pone.0257314.s010.docx]

Journal Requirements:

**Please review your reference list to ensure that it is complete and correct. If you have cited papers that have been retracted, please include the rationale for doing so in the manuscript text, or remove these references and replace them with relevant current references. Any changes to the reference list should be mentioned in the rebuttal letter that accompanies your revised manuscript. If you need to cite a retracted article, indicate the article’s retracted status in the References list and also include a citation and full reference for the retraction notice.**

Answer: We reviewed our reference list and we can confirm that it is complete and correct. None of the included reference was noticed as “retracted”.

[Note: HTML markup is below. Please do not edit.]

Reviewers' comments:

Reviewer's Responses to Questions

**Comments to the Author**

1. If the authors have adequately addressed your comments raised in a previous round of review and you feel that this manuscript is now acceptable for publication, you may indicate that here to bypass the “Comments to the Author” section, enter your conflict of interest statement in the “Confidential to Editor” section, and submit your "Accept" recommendation.

Reviewer #1: (No Response)

Reviewer #2: All comments have been addressed

2. Is the manuscript technically sound, and do the data support the conclusions?

The manuscript must describe a technically sound piece of scientific research with data that supports the conclusions. Experiments must have been conducted rigorously, with appropriate controls, replication, and sample sizes. The conclusions must be drawn appropriately based on the data presented.

Reviewer #1: Yes

Reviewer #2: Yes

3. Has the statistical analysis been performed appropriately and rigorously?

Reviewer #1: Yes

Reviewer #2: (No Response)

4. Have the authors made all data underlying the findings in their manuscript fully available?

The [PLOS Data policy](https://emea01.safelinks.protection.outlook.com/?url=http%3A%2F%2Fwww.plosone.org%2Fstatic%2Fpolicies.action%23sharing&data=04%7C01%7C%7C47e618b38043432dae3208d955c81f82%7C84df9e7fe9f640afb435aaaaaaaaaaaa%7C1%7C0%7C637635140275038489%7CUnknown%7CTWFpbGZsb3d8eyJWIjoiMC4wLjAwMDAiLCJQIjoiV2luMzIiLCJBTiI6Ik1haWwiLCJXVCI6Mn0%3D%7C1000&sdata=xonVbVR99klwLqKg39S31wh%2B970q8Uo4Qc4wDbtiZIo%3D&reserved=0) requires authors to make all data underlying the findings described in their manuscript fully available without restriction, with rare exception (please refer to the Data Availability Statement in the manuscript PDF file). The data should be provided as part of the manuscript or its supporting information, or deposited to a public repository. For example, in addition to summary statistics, the data points behind means, medians and variance measures should be available. If there are restrictions on publicly sharing data—e.g. participant privacy or use of data from a third party—those must be specified.

Reviewer #1: Yes

Reviewer #2: No

5. Is the manuscript presented in an intelligible fashion and written in standard English?

PLOS ONE does not copyedit accepted manuscripts, so the language in submitted articles must be clear, correct, and unambiguous. Any typographical or grammatical errors should be corrected at revision, so please note any specific errors here.

Reviewer #1: Yes

Reviewer #2: Yes

6. Review Comments to the Author

Please use the space provided to explain your answers to the questions above. You may also include additional comments for the author, including concerns about dual publication, research ethics, or publication ethics. (Please upload your review as an attachment if it exceeds 20,000 characters)

**Reviewer #1: Dear authors,

Thank you for your well-performed revision. I highly appreciated your efforts and feel that the manuscript has improved significantly. I only have a few minor comments left, most of them related to editing.

2.118 I agree with the content of the new sentence however the English could be improved**.

Answer: We improved the language of this sentence and we hope that now it is more appropriate.

Line 112: *As changes in P_va_CO_2_ may be affected by baseline value and as P_va_CO_2_ is inversely related to cardiac index, we separated patients into ‘low’ and ‘high’ cardiac index using a cut-off value of 2.2 L/min/m^2^, similarly to a previous study (15). Of note, the term ‘low CI’ should not be misinterpreted as in some case the low CI may still be adequate (20).’*

**11.257 The new line in the manuscript differs from the line given in the answers to the reviewers: I prefer the latter “****Fourth, we did not investigate thoroughly the effects of other therapeutic interventions (e.g. mechanical ventilation, inotropes) on PvaCO2 as well AS its changes during FB.”
This makes it clear that some conditions impacting on the correlation between central and mixed venous values might not only influence the effect of FB but the whole concept of PvaCO2.**

Answer: We agree with the reviewer and we changed this phrase to the text.

**Some content appears to be missing (Figure 3 and S3, S5 and S7)**

Answer: Our apologies for this inconvenience. We think that these contents are visible now.

**S8 Figure: I liked this tentative algorithm. However some ambiguity is introduced when using “<” and/or “>” in cases of negative changes (in this case in PvaCO2). Is a change of “-2” “<” or “>” compared to a change of “-5”? In the algorithm I would use “> -2 mmHg” but opinions probably differ on this. Perhaps words could be used instead.**

Answer: We thank the reviewer for the appreciation of the suggested algorithm and we agree that the mathematical expressions can cause misunderstandings. We replaced them with words.

**Reviewer #2: I think the authors addressed all the questions raised during the review process adequately.
I think the results section of the abstract needs to be rephrased to reflect the clear findings presented in the first paragraph of the discussion section. The abstract as it is now is still a little confusing.**

Answer: We rephrased the results section of the abstract. We hope now it is easier to be read.

**Also, I am not sure if the authors included S3 table, S5 figure, S7. I am not able to see them in the attached manuscript.**

Answer: Our apologies for this inconvenience. We think that these contents are visible now.

7. PLOS authors have the option to publish the peer review history of their article ([what does this mean?](https://emea01.safelinks.protection.outlook.com/?url=https%3A%2F%2Fjournals.plos.org%2Fplosone%2Fs%2Feditorial-and-peer-review-process%23loc-peer-review-history&data=04%7C01%7C%7C47e618b38043432dae3208d955c81f82%7C84df9e7fe9f640afb435aaaaaaaaaaaa%7C1%7C0%7C637635140275048446%7CUnknown%7CTWFpbGZsb3d8eyJWIjoiMC4wLjAwMDAiLCJQIjoiV2luMzIiLCJBTiI6Ik1haWwiLCJXVCI6Mn0%3D%7C1000&sdata=u0y8DwBuFDvxo61AcqueWbo22p88vEQ%2B8F2AXd107JI%3D&reserved=0)). If published, this will include your full peer review and any attached files.

Answer: We agree to publish the peer review history.


If you choose “no”, your identity will remain anonymous but your review may still be made public.


**Do you want your identity to be public for this peer review?** For information about this choice, including consent withdrawal, please see our [Privacy Policy](https://emea01.safelinks.protection.outlook.com/?url=https%3A%2F%2Fwww.plos.org%2Fprivacy-policy&data=04%7C01%7C%7C47e618b38043432dae3208d955c81f82%7C84df9e7fe9f640afb435aaaaaaaaaaaa%7C1%7C0%7C637635140275058403%7CUnknown%7CTWFpbGZsb3d8eyJWIjoiMC4wLjAwMDAiLCJQIjoiV2luMzIiLCJBTiI6Ik1haWwiLCJXVCI6Mn0%3D%7C1000&sdata=IFx6fE2XPQ5uRp%2BEz10k9kC%2FeAbO71xLEOM6MghWVbw%3D&reserved=0).

Reviewer #1: No

Reviewer #2: No

[NOTE: If reviewer comments were submitted as an attachment file, they will be attached to this email and accessible via the submission site. Please log into your account, locate the manuscript record, and check for the action link "View Attachments". If this link does not appear, there are no attachment files.]

While revising your submission, please upload your figure files to the Preflight Analysis and Conversion Engine (PACE) digital diagnostic tool, [https://pacev2.apexcovantage.com/](https://emea01.safelinks.protection.outlook.com/?url=https%3A%2F%2Fpacev2.apexcovantage.com%2F&data=04%7C01%7C%7C47e618b38043432dae3208d955c81f82%7C84df9e7fe9f640afb435aaaaaaaaaaaa%7C1%7C0%7C637635140275058403%7CUnknown%7CTWFpbGZsb3d8eyJWIjoiMC4wLjAwMDAiLCJQIjoiV2luMzIiLCJBTiI6Ik1haWwiLCJXVCI6Mn0%3D%7C1000&sdata=tLrBJOCyWZTd8jy6Khd%2BEc68TqaGFkKk8SENrKhOy2Q%3D&reserved=0). PACE helps ensure that figures meet PLOS requirements. To use PACE, you must first register as a user. Registration is free. Then, login and navigate to the UPLOAD tab, where you will find detailed instructions on how to use the tool. If you encounter any issues or have any questions when using PACE, please email PLOS at [figures@plos.org](mailto:figures@plos.org). Please note that Supporting Information files do not need this step.
